# Cloning, expression and characterisation of a novel mollusc α-1,2-Fucosyltransferase from *Crassostrea gigas* (*Cg*FUT2)

**DOI:** 10.1007/s10719-024-10162-x

**Published:** 2024-08-20

**Authors:** Marilica Zemkollari, Colin Ruprecht, Markus Blaukopf, Reingard Grabherr, Erika Staudacher

**Affiliations:** 1 Department of Chemistry, University of Natural Resources and Life Sciences, Vienna, Austria; 2 Department of Biotechnology, University of Natural Resources and Life Sciences, Vienna, Austria

**Keywords:** α-1,2-fucosyltransferase, *Crassostrea gigas*, N-glycans

## Abstract

Glycans containing fucose play crucial roles in cell biology, particularly in recognition processes. In humans, fucose found in H-blood group antigens is recognized by various pathogens, thereby influencing host-pathogen interactions. However, in invertebrate biology the specific functions of these modifications and the corresponding glycosyltransferases are not fully elucidated. Therefore, cloning these glycosyltransferases from different model systems will provide valuable insights into this process. Little is known about fucosyltransferases in molluscs. For this study, a sequence of the Pacific oyster, *Crassostrea gigas*, based on amino acid sequence homologies with rabbit and human α-1,2-fucosyltransferases, was chosen. The recombinant enzyme (350 amino acids) was able to transfer fucose from GDP-fucose to the galactose residue of type II disaccharides, terminal galactoses in complex N-glycan structures and several linear and branched galactans which were tested using a glycan microarray. The α-1,2-linkage formed was confirmed by NMR analysis. The enzyme was active in a broad pH-range, it was relatively stable upon storage conditions and its activity was not dependent on the presence of divalent cations. In this study, we were able to clone, express and characterise a novel α-1,2-fucosyltrasferase from *Crassostrea gigas* (*Cg*FUT2).

**Supplementary Information:**

The online version contains supplementary material available at 10.1007/s10719-024-10162-x.

## Graphical Abstract

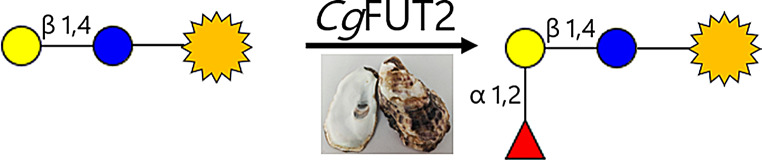

## Introduction

Fucosylated glycoconjugates play a number of biological roles in living organisms, including for example host pathogen recognition that may be mediated by carbohydrate-binding proteins. Fucose can be found in different linkages (α-1,2-, α1,3-, α-1,4-, and α-1,6-) with other sugars. The most common type of fucose modification is the fucosylation of the innermost GlcNAc in the core N-glycans structure by an α1,6- linkage [1]. Terminal fucosylation is another type of fucose modification. It is found on N-glycans, O-glycans, glycolipids and consists of fucosylation of terminal galactoses (α-1,2-) and subterminal N-acetylglucosamines (α-1,3/4-). FUT1 and FUT2 are the two fucosyltransferases which are responsible for transferring fucose to terminal galactose in α-1,2- linkage (EC 2.4.1.69) and thus the synthesis of the H-antigen [2, 3]. FUT1 is known as the H-transferase and is expressed in the red blood cell precursors synthesising the H-antigen, while FUT2, also is known as the secretor (Se)-transferase, generating H-antigen in body secretions such as saliva. In humans, the lack of functional gene copies of these two enzymes causes the special Bombay phenotype [4]. α-1,2- fucosyltransferases are also critically involved in many different types of cancer [5–8], fertility and pregnancy [9–13], and impact susceptibility to different parasites [14, 15].

FUT1 and FUT2 have been well studied in mammals, mostly humans, due to their crucial impact in various biological processes. Yet, their role in invertebrates, especially in molluscs, is far from being elucidated. Molluscs are vital components of many different ecosystems. They inhabit terrestrial, marine and fresh water habitats. Molluscs are filter feeders, improving the quality of water, they are decomposers and serve as food source for many other species, including humans. Gastropods that feed on plants are serious threats in agriculture [16]. Finally, many molluscs serve as intermediate host of human or cattle parasites, such as *Biomphalaria glabrata* for the trematode *Schistosoma mansoni* [17]. *Crassostrea gigas*, the Pacific oyster, is a bivalve native to the Pacific coast of Asia. In the last decades it has gained significant attention in the world of aquaculture and is currently being cultivated worldwide [18–24]. Together with its significant ecological value in improving the health of marine ecosystems and its economic value as a culinary delicacy, latest studies have shown that *C. gigas* can also be a suitable model organism to study human diseases [25]. Furthermore, this oyster is involved in the transmission of noroviruses and in bioaccumulation of pathogens [26]. Identifying glycosyltransferases in *C. gigas* can shed light on the evolutionary history and their adaptations to different ecological niches. Specific findings may also have implications for aquaculture, food technology and human health.

Only few α-1,2-fucosyltransferases from invertebrate origin have yet been described, two from *C. elegans* (CE2FT-1 and CE2FT-2) [27, 28] and two from mollusc origin, from the snail *L. stagnalis* [29] and recently a FUT1 from *Crassostrea gigas* [30]. These enzymes show high diversity and low sequence similarities compared with vertebrate enzymes.

The aim of this study was to expand the knowledge about the potential of mollusc glycobiology. Due to their diverse glycan structures, these organisms are highly interesting in terms of evolution and biodiversity, they might be suitable as model systems or even as production systems for specifically glycosylated proteins. Here we present the coning, expression and characterisation a novel α-1,2- fucosyltransferase (*Cg*FUT2) from the Pacific oyster *Crassostrea gigas*. This enzyme catalyses the transfer of a fucose from GDP-fucose to a terminal linked galactose forming glycans which are known to have a high impact in in vivo recognition processes.

## Materials and methods

### Materials

Restriction enzymes, T4 ligase, Q5 DNA polymerase and OneTaq DNA Polymerase were all purchased from New England Biolabs (Frankfurt, Germany). Plasmid purification and Gel clean-up kits were purchased from Macherey-Nagel (Düren, Germany). The g-Block gene fragment was synthetized by Integrated DNA Technologies (Leuven, Belgium). All primers were commercially synthetized by Integrated DNA Technologies or Sigma Aldrich (Vienna, Austria).

Chemically competent *Escherichia coli* (High Efficiency) cells (New England Biolabs, Frankfurt, Germany), DH10 Multibac YFP *E. coli* cells (Geneva Biotech, Geneva Switzerland) and *Spodoptera frugiperda* cells (Sf9, ATCC CRL-1711, Manassas Virginia) were cultivated in IPL41 medium (HyClone Cytiva—Vienna, Austria) were used.

Para-nitrophenol labelled mono- and disaccharides were obtained from Merck (Vienna, Austria).

### Identification of the gene sequence

The protein sequence of the α-1,2-fucosyltransferse from *Crassostrea gigas* was obtained by performing a blastP search, using the sequence of the rabbit (NP_001075871.1) and the human (NP_000502.4) α-1,2-fucosyltransferse as template. They were codon optimized for insect cells and synthetized by Integrated DNA Technologies (Leuven, Belgium).

### Cloning and expression of the synthetic sequences

The optimized gene sequences were amplified with primers containing 6HisTag (C-terminal) and EcoRI or XbaI restriction digestion sites for directional insertion into the pACEBac1 vector (5’ GATGAT GAATTC ATG CAA GAT TTC TTG AGG AAA TTT ACC and 5’ GATGAT TCTAGA TTA GTG ATG GTG GTG GTG GTG TGA ACC TGA GCC TGA TCA CAT CGG TAT CCA CTG CG). The clones were verified by DNA sequencing. Correct clones were integrated in the Baculovirus genome via Tn7 transposition in DH10EMBacY cells (Geneva Biotech, Genève, Switzerland). The Bac-To-Bac Baculovirus expression system was used to express the enzymes in Sf9 insect cells as previously described [31]. The cells were lysed in MES buffer pH 7.0 using an Ultraturrax. This preparation was used for further analysis, because any purification attempt (see below) failed.

Sf9 cells expressing *Cg*FUT2 were lysed in Tris buffer (pH 7.0), and the supernatant was used for purification. Protein A/G agarose beads (100 µL, CALBIOCHEM, San Diego, United States) coupled with mouse anti-Penta Histidine Tag monoclonal antibody (BIORAD, Vienna, Austria) were utilized. The beads were incubated with the anti-Penta Histidine Tag antibody for 1 h at room temperature with continues rotation. The antibody coupled beads were incubated with the cell lysate (1 mL) at room temperature for 1 h with continuous rotation. Following incubation, the beads were centrifuged, the supernatant was removed, and the beads were washed five times with TBS buffer. Subsequently, the beads were resuspended in MES buffer (pH 7.0, 100 µL), analysed by SDS-PAGE and tested for activity.

hFc tag: the *Cg*FUT2-pACEBac1 sequence and the hFc tag sequence (from pYD11 plasmid) were amplified with primers containing SalI or XbaI restriction digestion sites to ensure directional insert of the hFc tag in the *Cg*FUT2-pACEBac1 construct. The following primers were used: 5’ GATGAT GTCGAC GAA AAC CTG TAT TTT CAG GGC ACT CAC and 5‘GATGAT TCTAGA TTA TTT CCC GGG AGA CAG GGA (hFc tag) and 5’ GATGAT GTCGAC GTA ATG GTG GTG GTG GTG ACC TGA GCC TGA CAT GCC and 5’GATGAT TCTAGA TCT AGA GCC TGC AGT CTC G (to linearize the *Cg*FUT2-pACEBac1 construct). Sf9 cells expressing this construct were lysed in Tris buffer (pH 7.0). Protein A/G agarose beads were used for purification (as described above).

Maltose Binding Protein Tag: the *Cg*FUT2-pACEBac1 sequence and the MBP tag sequence (from pMAL-c2E plasmid) were amplified with primers containing EcoRI and SalI restriction digestion sites. The following primers were used: 5‘ATATCT GTCGAC ATG AAA ACT GAA GAA GGT AAA CTG GTA ATC TGG and 5’GATGAT GAATTC CTT GTC ATC GTA ATC CCC GAG GTT GTT (to amplify the MBP sequence) and 5’GATGAT GAATTC ATG CAA GAT TTC TTG AGG AAA TTT ACC CTC and 5’ ATA TCT GTCGAC CGC GCG CTT CGG AC (to linearize *Cg*FUT2-pACEBac1 construct). Sf9 cells expressing this construct were lysed in Tris buffer (pH 7.0). Amylose resin (New England Biolabs, Frankfurt, Germany) was used for purification. 1mL of Sf9 cell lysate was incubated with 500 µL of resin slur for 2 h. The resin was washed 5 times with column buffer (New England Biolabs, Frankfurt, Germany) and the enzyme was eluted with maltose (New England Biolabs, Frankfurt, Germany). The eluted sample was analysed by SDS-PAGE and tested for activity.

### Activity assay of recombinant CgFUT2

The activity of the recombinant α-1,2-fucosyltransferse was measured in a total volume of 20 µL reaction mixture containing: 100 mM MES pH 7.0, 20 mM MnCl_2_, 3 mM GDP-Fuc (Sigma-Aldrich, Vienna, Austria), 0.5 mM pNP-β-lactose, 2 mM ATP and 13 µL of cell lysate (approximately 20 µg of total protein) at 37 °C for 2 h. The reaction was terminated by cooking the sample at 100 °C for 5 min and analysed by HPLC on a reversed phase C18 column 4 × 250 mm, 5 μm (Thermo Scientific, Vienna, Austria) in 0.1 M ammonium acetate pH 6.0 (solvent A) applying a linear gradient with solvent B (acetonitrile in water 50:50) from 5 to 50% in 30 min at a flow rate of 1 mL/min with a detection at 280 nm.

### NMR analysis

NMR spectra were recorded with a Bruker Avance III 600 instrument (600.22 MHz for 1H, 150.93 MHz for ^13^C) using standard Bruker NMR software. ^1^H spectra were referenced to 0.00 (D_2_O, external calibration to 2,2-dimethyl-2-silapentane-5-sulfonic acid) ppm unless stated otherwise. ^13^C NMR spectra were referenced to 67.40 (D_2_O, external calibration to 1,4-dioxane) ppm. Structure assignments were based on COSY, HSQC, HMBC and TOCSY data.

### Substrate specificity

Substrate specificity was tested with the artificial monosaccharide substrates pNP-α-Gal, pNP-α-Glc, pNP-β-GalNAc, pNP-β-Gal, pNP-β-Glc, pNP-β-GlcNAc and pNP-lactose (commercially obtained, Merck, Vienna, Austria) and analysed by HPLC as described in Sect. 2.5. Sugars labelled with anthranilic acid (AA) (lactose, lacto-N-biose, galacto-N-biose, N-acetylactosamine, Galβ1,6GalNAc) were prepared as previously described [32] and analysed on reverse-phase HPLC C18 column ODS HypersilTM, 250 × 4 mm, in 0.2% (v/v) 1-butylamin, 0.5% (v/v) orthophos-phoric acid, 1% (v/v) tetrahydrofuran in H2O (solvent A) and 50% solvent A in acetonitrile (solvent B), applying a linear gradient of solvent B from 5 to 100% in 23 min, at a flow rate of 1 mL/min, with fluorescence detection at Ex/Em 360 nm/425 nm. 2-Aminopyridine (PA) labelled substrates (lactose-PA; Galβ1,4-GlcNAcβ1,2Manα1,6 (Galβ1,4-GlcNAcβ1,2Manα1,6)Manβ1,4GlcNAcβ1,4GlcNAc-PA, GalGal-PA; Glc-NAcβ1,2Manα1,6 (GlcNAcβ1,2Manα1,6)Manβ1,4GlcNAcβ1,4GlcNAc-PA, GnGn-PA) were prepared as previously described [33] and analysed by HPLC on a reverse phase C18 column, 250 × 4 mm, in 0.1 M NH4Ac pH 4.0 (solvent A) applying a linear gradient with solvent B (30% methanol in water) from 0 to 30% in 30 min, with a flow rate of 1.5 ml/min with fluorescent detection at Ex/Em: 320 nm/400nm.

The glycan array was performed as previously described [34]. In brief, 90 µl of enzyme solution was mixed with 10 µl of a 1 mM GDP-azido-fucose (biotechne RnD systems) and applied to the glycan microarray slide using a grid with 16 wells. After incubation for 16 h, the slide was washed twice with PBS for 5 min, the grid was removed and the slide was further washed three times with PBS containing 1% SDS for 15 min under continuous shaking. Next, the click reaction was carried out on the slide using a 16-well grid. For this, a 1:1 DMF: water mixture, including the Sulfo-Cy5 dye (Jena Biosciences, 2mM final concentration), premixed CuSO_4_ (1 mM) and tris(3-hydroxypropyltriazolylmethyl)amine (THPTA, Roth, 1 mM), and sodium ascorbate (10 mM), was incubated for 1 h on the array. Next, the grid was removed and the slide was washed three times with 1% SDS in PBS for 20 min to remove unreacted dye, then two times with deionized water for 5 min to remove salts, and finally dried and scanned for fluorescence using a microarray scanner (Molecular Devices). Images were recorded at 300 PMT gain and processed using the GenePix Pro 7 software (Molecular Devices). Oligo-saccharides were chemically synthesized by solution phase synthesis (73–92 [35–37]) or automated solid phase synthesis (26–68, [38–40]). A cell extract without expressed enzyme, as well as a cell extract with a different enzyme expressed, served as appropriate controls.

### Biochemical parameters

For the analysis of the biochemical parameters the standard assay conditions were modified as follows. For the determination of cation requirements, the assay was carried out without any cations or in presence of 10 mM EDTA, Mn^2+^, Mg^2+^, Ca^2+^, Ba^2+^, Co^2+^, Cu^2+^ or Ni^2+^. The determination of enzyme stability and pH optimum was done according to [31] Inhibition experiments were carried out in the presence of 0,25 mM UMP, UDP, UTP, GDP, Gal, GalNAc or Glc to the standard incubation assay. For the temperature storage assays, aliquots of 30 µL of enzyme were stored at different temperatures and one aliquot was analysed at each time point. While for the freeze and thaw cycles a 500 µL aliquot was stored at -20 °C and another one at -80 °C. At each time point the same aliquot was thawed and frozen again, giving in total 8 freeze/thaw cycles over a period of 4 weeks.

Each assay was performed in several biological replications, each at least in duplicate, with appropriate controls.

### Matrix assisted laser desorption Ionisation—time of flight (MALDI-TOF) mass spectrometry

MALDI-TOF MS analysis was carried out on an Autoflex Speed MALDI-TOF (Bruker Daltonics, Germany) equipped with a 1000 Hz Smartbeam.II laser in positive mode using dihydroxybenzoic acid as matrix (2% w/v in 50% v/v acetonitrile solution). For crystallization 1 µL of an 1:40 dilution of the samples was spotted on the plate, air dried, covered by 1 µL of matrix solution and again air dried. Spectra were processed with the manufacturer’s software (Bruker Flexanalysis 3.3.80).

## Results

### Identification, cloning and expression of CgFUT2

**Fig. 1:**
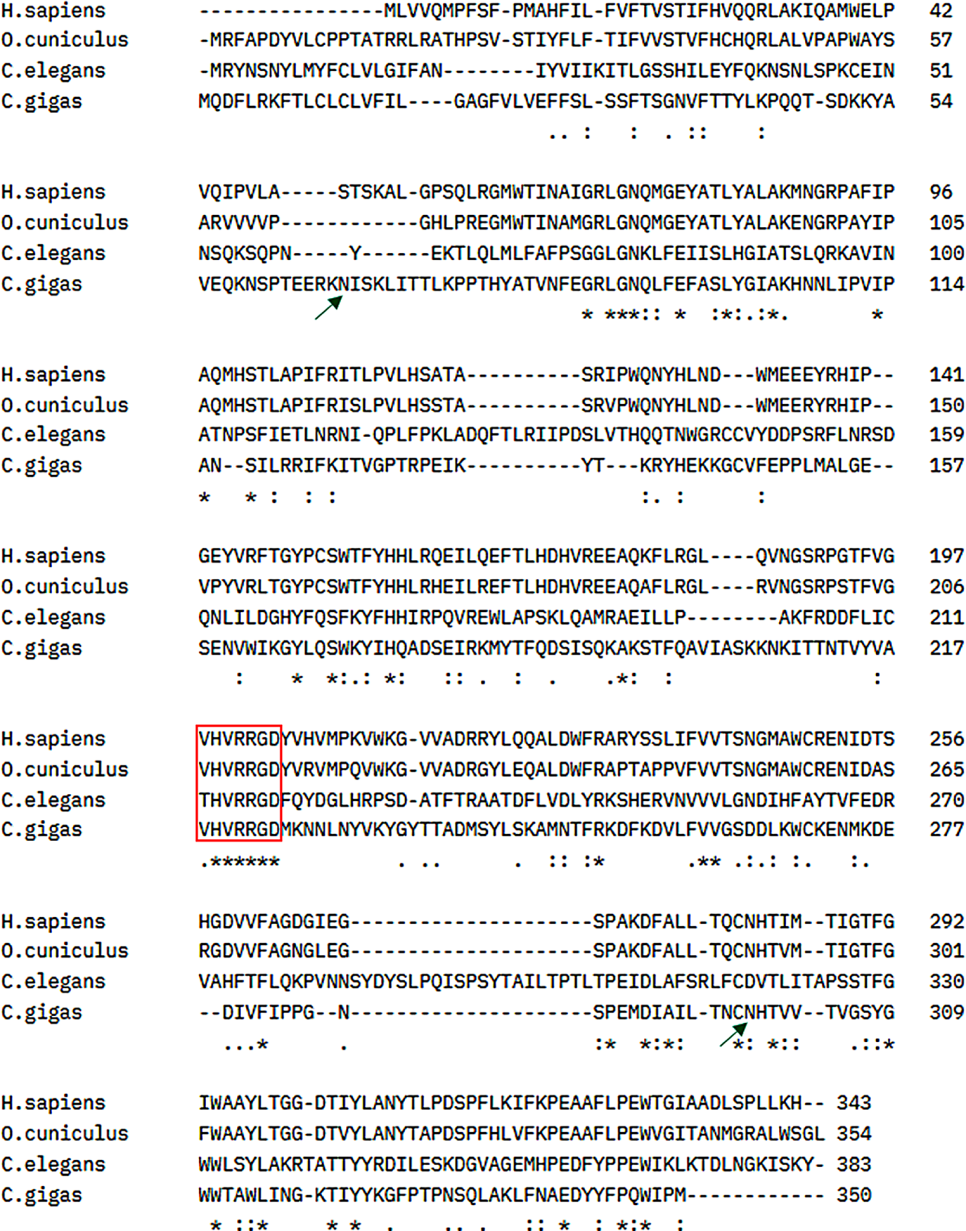
Sequence alignment of α-1,2- fucosyltransferase from *H. sapiens* (NP_000502.4), *O. cuniculus* (NP_001075871.1), *C. elegans* (ABK20307.1) and *C. gigas* (WNI04759.1). Conserved domains are showed in red box. Putative N-glycosylation sites are indicated by green arrows. (* fully conserved residues,: residues with strongly similar properties,. residues with weakly similar properties, - indicates gap). Sequence alignments were prepared using CLUSTAL O (1.2.4)

The gene sequence used for this study was selected based on sequence homologies to rabbit (NP_001075871.1), human (NP_000502.4) and *C. elegans* (ABK20307.1) α-1,2-fucosyltransferases. The identified coding sequence has 1053 nucleotides and has low similarities with the previously described enzymes from other species: rabbit 30.85%, human 31.5%, *C. elegans* 25.90% and *Cg*FUT1 35,64%. The protein has an approximate molecular weight of 41.4 kDa containing 350 aa. It is a type II transmembrane Golgi protein (transmembrane domain region: residues 14–32, predicted by TMHMM Server v. 2.0) with two putative N-glycosylation sites. It contains the amino acid motif VHVRRGD, which is conserved throughout fucosyltransferases, with the two arginines at position 221 and 222 that bind GDP-fucose [41] (Fig. 1).

The *Cg*FUT2 was cloned into three different vectors to obtain protein fusions with a 6xHisTag, a hFc tag or a Maltose-binding Protein (MBP) tag at the C-terminus. These three constructs were suitable for expression of *Cg*FUT2 in insect cells (Sf9) using a Bac-to-Bac Baculovirus expression system. Unfortunately, the purification failed in all three cases due to lack of binding of *Cg*FUT2 to anti HisTag antibody beads (His Tag construct) or insufficient protein yield and activity (hFc tag) or poor purity of *Cg*FUT2 after purification (MBP tag). Therefore, the crude cell lysate was used for further biochemical analyses. The enzyme activity correlated in a linear manner to the amount of cell lysate used in the assay.

### Substrate specificity determination

The activity of Sf9 cell lysate expressing *Cg*FUT2 and a control Sf9 cell lysate was assessed using pNP-β-lactose as the substrate (Fig. S1). The cell lysate expressing α-1,2-fucosyltransferase displayed high activity levels, while the control cell lysate showed no activity.

NMR analysis of the purified HPLC product confirmed the α-1,2 linkage of fucose to the galactose residue of the substrate. Interestingly, upon comparison to the starting material pNP-lactose, a new signal in the ^1^H NMR anomeric region was detected at 5.29 ppm (Fig. S2) and a low field shift corresponding to the anomeric galactose signal could be observed. HSQC spectra further showed that the correlated carbon shift for this new signal suggested a new anomeric signal (102.29 ppm). Additionally, a strong indicative signal for the fucose methyl moiety was found at 1.21 ppm, implying the presence of an additional fucose. The *J*_H1_,_H2_ coupling was found to be 2.7 Hz for the signal at 5.29 ppm and the *J*_H, C_ coupling of this anomeric signal was found via CLIP HSQC to be 172 Hz. Therefore, the anomeric configuration of the introduced fucose was determined to be alpha.

The additionally detected anomeric signal showed a notable HMBC correlation to position 2 of the galactose moiety. Furthermore C 2 of galactose showed a noticeable ^13^C shift as compared to the starting material pNP-lactose from 74.1 to 78.5 ppm (Fig. S2). It was therefore concluded that the obtained product was p-nitrophenyl-O-α-L-fucopyranosyl-(1→2)-β-D-galactopyranosyl (1→4)-D-glucopyranoside. These findings were in full agreement with published data [42].

**Fig. 2:**
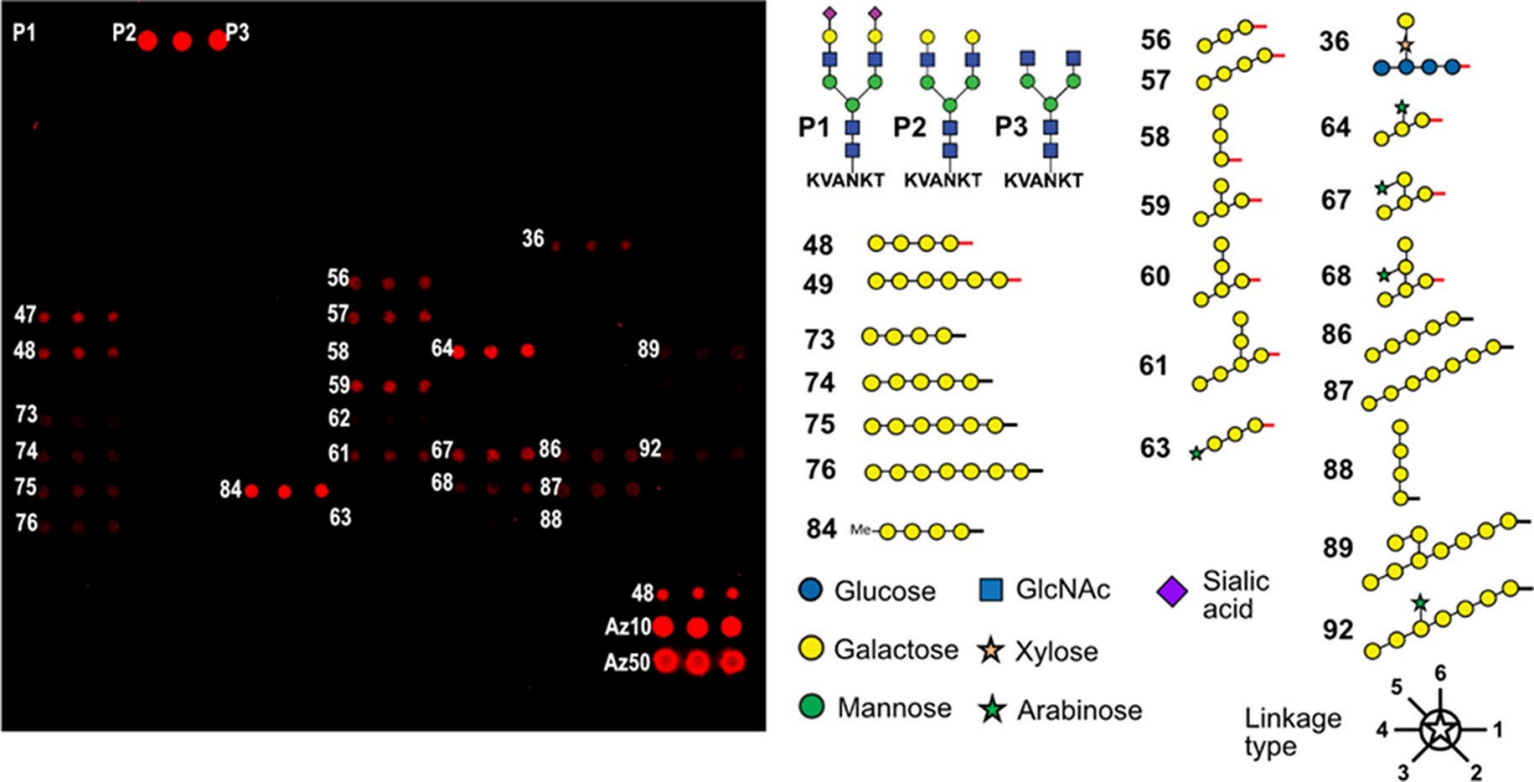
Analysis of the substrate specificity of *Cg*FUT2 using a glycan microarray. Annotated are only substrates of *Cg*FUT2 and a few oligosaccharides that are not substrates but mentioned in the text. The angle of the linkage between sugar symbols indicates the type of linkage between the sugars. P1-P3 are N-linked glycopeptides isolated from egg yolk. The red and black bars denote different linkers used for immobilization of the glycans on the array. For more information and a complete list of compounds on the microarray slide see Fig. S5

To explore the substrate specificity of *Cg*FUT2, various p-nitrophenol-labelled monosaccharides (pNP-α-Gal, pNP-α-Glc, pNP-β-GalNAc, pNP-β-Gal, pNP-β-Glc, pNP-α-GlcNAc, and pNP-β-GlcNAc) and disaccharides labelled with anthranilic acid (AA) (lacto-N-biose, galacto-N-biose, N-acetylactosamine and Galβ1,6GalNAc) were tested using HPLC separation. However, none of those was a suitable substrate. 2-Aminopyridine labelled N-glycans (GalGal-PA and GnGn-PA) were tested using MALDI-TOF mass spectrometry. GalGal-PA turned out to be a suitable substrate (Fig. S3). Lactose was tested with three different markers to test whether the labelling had any influence on enzyme activity. No difference in the transfer rate was found between pNP-lactose, AA-lactose and PA-lactose (Fig. S4). To further investigate the substrate specificity of *Cg*FUT2, we used a recently developed glycan array-based method for the characterization of glycosyltransferases [34]. In this assay, *Cg*FUT2 was incubated with an azido-modified GDP-fucose on a glycan microarray that was equipped with about 100 oligo- and polysaccharides, including three different purified N-glycopeptides and many chemically synthesized oligosaccharides related to plant cell wall polysaccharides. During the reaction, *Cg*FUT2 was specifically attached to azido-fucose to appropriate oligosaccharide acceptor molecules. Next, the azide was coupled to a fluorescent dye, in this case Cy5, using copper-catalysed click chemistry and the fluorescence signal was detected using a microarray scanner (see Fig. S5a for assay principle). This experiment provided a direct readout of the substrate specificity of *Cg*FUT2. As expected, the glycopeptide with the GalGal N-glycan (P2) was the acceptor molecule, resulting in the highest fluorescence signal (Fig. 2, Table S1).

We noticed that also several plant cell wall related oligosaccharides were substrates with intermediate fluorescence signal, including linear and branched galactans of a minimal length of at least three monosaccharides. In this case apparently the linker to the array had an effect. A glycan connected to a longer linker (for example compounds 48 and 49) gave a more intense signal than the same glycan connected to a shorter linker (compound 73 and 74). Additionally, xyloglucan oligosaccharides with a terminal galactose substitution were found to be suitable substrates (compound 64, with lower signal also compound 67), suggesting a certain acceptor substrate promiscuity of *Cg*FUT2, tolerating also monosaccharides other than GlcNAc adjacent to the fucosylated galactose. These monosaccharides could be galactoses in β-1,3- or β-1,4-linkage (e.g. compounds 56 and 57 and 48 and 49), or xylose in a β-1,2 linkage (compound 36), but not a galactose in β-1,6-linkage (for example compounds 58 and 88). Analysing a possible substitution of the acceptor showed that methylation in the O4-position tended to improve the fucosylation of a galactan tetrasaccharide (compare compounds 84 and 73), while a fluorine in the same position prevented the transfer (compound 85). A substitution in O3-position of the galactose by sialic acid or arabinose (compare glycopeptide P1 and P2 or oligosaccharide 56 and 63, respectively) abolished the fucosylation by *Cg*FUT2. Xylan and glucan-related oligosaccharides did not show incorporation of fucose, corroborating that only terminal galactoses are fucosylated by *Cg*FUT2 (see Fig. S5 for the complete set of tested compounds on the glycan microarray). It is important to note that this glycan microarray-based method is not quantitative and only relative acceptor substrate preferences of the analysed glycosyltransferase can be concluded.

### Biochemical properties of the enzyme

**Fig. 3:**
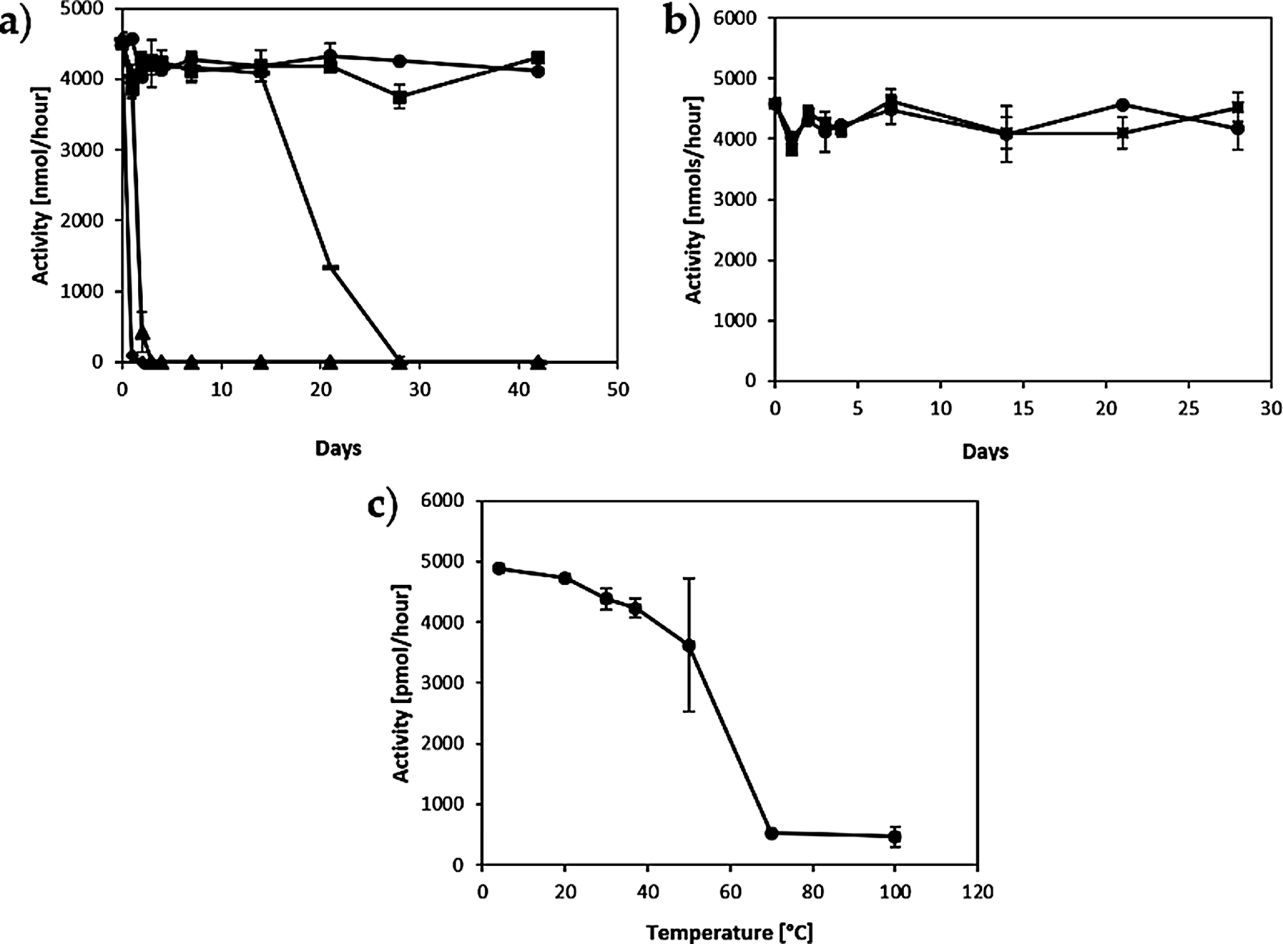
Effects of temperature on CgFUT2 activity **a**) Storage temperature ( ▪ -80°C •-20°C, - 4°C, ▴ 20°C, ♦ 37°C), **b**) thaw and freeze cycles and c) incubation temperature

**Fig. 4:**
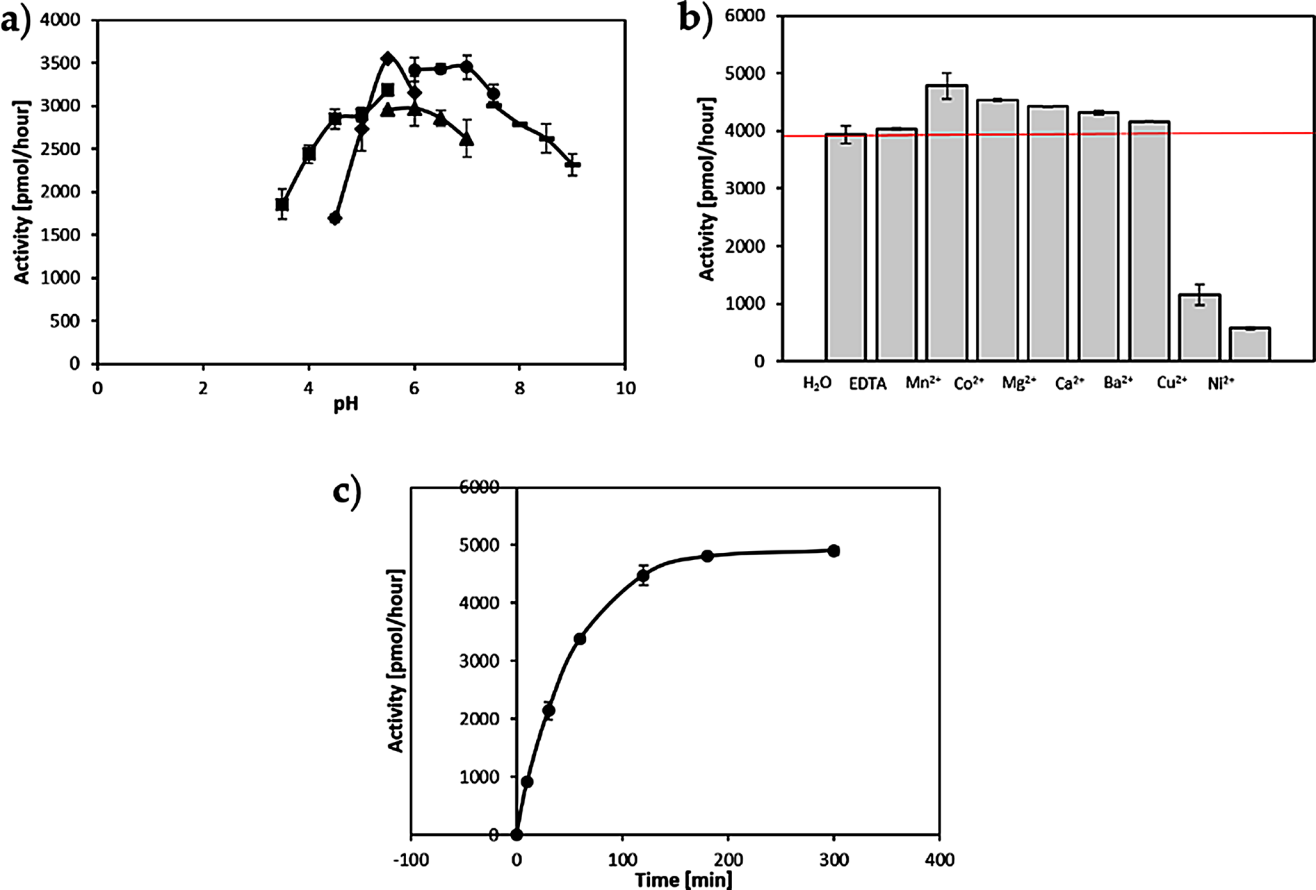
Effects of **a**) pH ( ▪ Acetate, ♦ Citrate, ▴ MES, • Phosphate, - Tris), **b**) divalent cations (for better comparison a line shows the level of transfer without addition of a cation) and c) incubation time on CgFUT2 activity

The *Cg*FUT2 activity was found to be relatively stable upon storage at temperatures ranging from − 80 °C to 37 °C for 24 h. The activity of the aliquots stored at 37 °C and 20 °C was abolished after 48 h and 72 h respectively. The aliquots stored at 4 °C completely lost the activity at week 4, while the aliquots stored at -20 °C and − 80 °C did not show significant changes in the activity rates even after 6 weeks of storage (Fig. 3a). Thaw and freeze cycles did not affect the activity either (Fig. 3b). The optimal incubation temperature was 15–20 °C (Fig. 3c) and the transfer was linear up to 50 min (Fig. 4c).

Maximal rates of transfer were observed in citrate buffer pH 5.5. In general, the activity of the enzyme was high at pH ranging from 5.0 to 8.0 in all the four different buffers tested (Fig. 4a). The enzymatic reaction of the *Cg*FUT2 was not dependent on divalent cations, as observed in the standard activity assay without any cations and in the presence of 20 mM EDTA, Mn^2+^, Mg^2+^, Ca^2+^, Co^2+^, Cu^2+^, Ni^2+^, or Ba^2+^. The presence of EDTA did not abolish the transfer activity. Addition of manganese ions increased the activity by 15%. Addition of Mg^2+^, Ca^2+^, Co^2+^ and Ba^2+^ had slightly positive effects on the activity, while presence of Cu^2+^ and Ni^2+^ drastically decreased the activity (Fig. 4b).

**Fig. 5:**
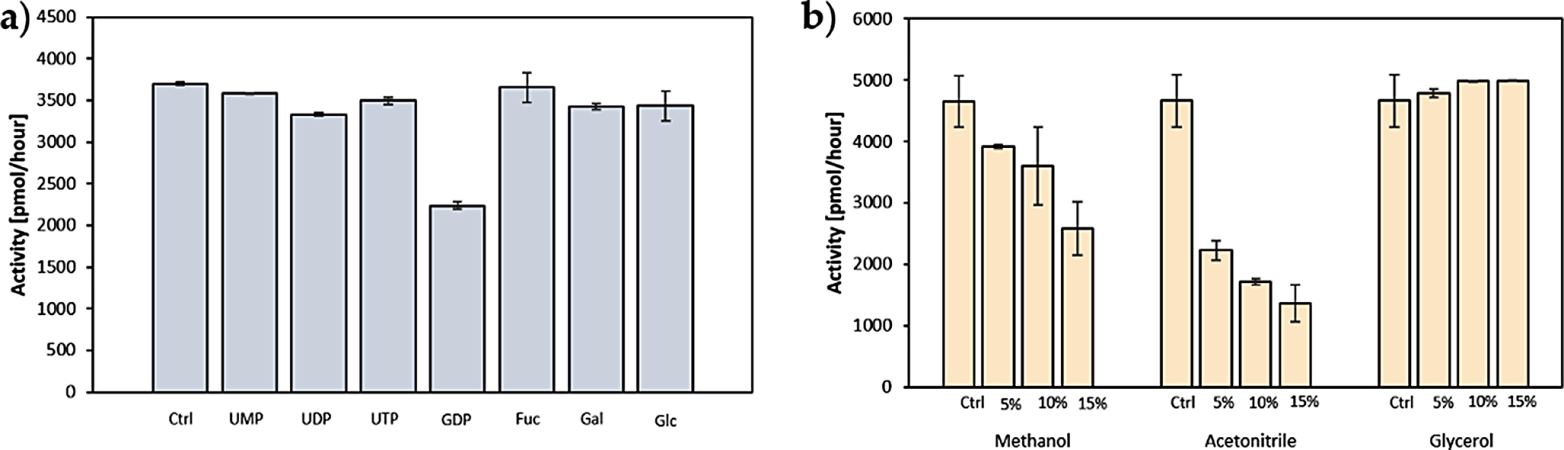
Effects of different compounds on *Cg*FUT2 activity **a**) nucleotide phosphates and sugars and **b**) methanol, acetonitrile and glycerol (Ctrl, control, standard assay conditions)

The presence of 0,25 mM UMP, UDP, UTP, fucose, glucose and galactose had no effect on the activity, while the presence of 0,25 mM GDP decreased the activity by 30% (Fig. 5a). Addition of up to 15% of glycerol had no effect on the activity of the enzyme. Presence of 5%, 10% and 15% of methanol decreased the activity by 14%, 21% and 41% respectively. Acetonitrile had the highest impact on the activity as addition of 5%, 10% and 15% of it decreased the activity by 48%, 58% and 65% respectively (Fig. 5b).

## Discussion

In this study we describe the molecular cloning and biochemical characterisation of an α1,2-fucosyltransferase from the Pacific oyster, *Crassostrea gigas* (*Cg*FUT2). The cDNA sequence predicted a 350 amino acid protein, with a transmembrane domain, which is expected for all Golgi glycosyltransferases.

*Cg*FUT2 showed low amino acid sequence similarities to previously described enzymes from other species: rabbit 30,85%, human 31,5%, *C. elegans* 25,90%. This is not surprising, since the degree of identity between fucosyltransferases is generally low. The *C. elegans’* α-1,2-fucosyltransferase has only 5–10% identity to respective mammalian enzymes [27]. This diversity in sequence is often associated with different substrate specificities, allowing these enzymes to perform distinct roles in different biological contexts. This makes characterisation based on sequence comparison rather difficult. The only motif they have in common is their GDP-fucose binding site. A conserved peptide motif within all α 1,2- and α 1-6-fucosyltransferases has been identified [41]. The *Cg*FUT2 contains the motif VHVRRGD, where the two arginines at position 221 and 222 directly bind to GDP-fucose. The rest of the sequence does not show any conserved regions when compared other previously described α-1,2-fucosyltransferases (Fig. 1).

*Cg*FUT2 transferred fucose exclusively to terminal galactose residues. It fucosylated exclusively type II (Gal-β1,4-GlcNAc) disaccharide substrates and showed no activity against type I (Galβ1,3GlcNAc) or type III (Galβ1,3GalNAc) disaccharide substrates. The *Cg*FUT2 was able to fucosylate β-1,3-linked galactose chains of a certain length, as well as terminal β-1,2-linked or β-1,4-linked galactoses in glycans. No transfer was obtained using phenyl-β-Gal, any pNP-monosaccharide or β-1,6-linked galactoses. Several previously described α1,2-fucosyltransferases were able to transfer fucose to phenyl-β-Gal, with the exception of the CE2FT-2 [28]. The activity of *Cg*FUT2 showed a broad pH-optimum (5.0–8.0), but no preferences regarding the buffer salt. Further, the activity was not depending on divalent cations. High activity rates in a broad pH-optimum with various buffers and a wide range of acceptor substrates makes *Cg*FUT2 a good candidate for industrial utilisation like food industry and production of glycoconjugates for vaccine production.

Biochemical parameters for previously described invertebrate α-1,2-fucosyltrasferases were not determined, thus, it is not possible to compare them to *Cg*FUT2. However, we can compare the activity assays described in the corresponding publications. *C. elegans* α-1,2-fucosyltrasferase was tested in 100 mM sodium cacodylate pH 7.0, in the presence of 20 mM MnCl_2_, rabbit α-1,2-fucosyltrasferase was tested in 25 mM sodium cacodylate pH 6.1 without MnCl_2_, the pig enzyme was tested in 125 mM sodium phosphate without manganese ions and the corresponding human enzyme was tested in 25 mM sodium phosphate pH 6.0 also without MnCl_2_ [27, 28, 43, 44]. While *C. gigas* FUT1was tested in 100 mM Tris buffer pH 7.5 and 20 mM MnCl_2_ [30]. These variations in the activity assays of other invertebrate enzymes suggest that the pH optimum for these enzymes also lies within a broad range with no preferences of the buffer salt. Since none of these homologous α-1,2-fucosyltrasferases were tested in presence of EDTA, it is not clear whether divalent cations were required for activity of these enzymes or not.

The identification of *Cg*FUT2 (this paper) and *Cg*FUT1 [30] in oyster demonstrates that molluscs can synthetize H-blood group antigens. These structures are known to bind human noroviruses [45] that cause outbreaks of gastroenteritis. Since oysters are filter feeders, they accumulate these pathogens and can serve as vectors of noroviruses. The presence of structures similar to the human H-blood group antigen have been identified in oysters’ gastrointestinal tract [46–48], suggesting that binding of the norovirus in the oysters’ gut is mediated by fucosylated structures. This confirms the importance of characterising FUT1 and FUT2 from mollusc origin.

## Electronic supplementary material

Below is the link to the electronic supplementary material.

Supplementary Material 1

## Data Availability

Sequence data that support the findings of this study have been deposited in the NCBI data bank with the accession code WNI04759.1.

## References

[1] Schneider, M., Al-Shareffi, E., Haltiwanger, R.S.: Biological functions of fucose in mammals. Glycobiology. **27**, 601–618 (2017). 10.1093/glycob/cwx034 28430973 10.1093/glycob/cwx034 PMC5458543

[2] Lowe, J.B.: 7 the blood group-specific human glycosyltransferases. Baillieres Clin. Haematol. **6**, 465–492 (1993). 10.1016/S0950-3536(05)80155-6 8043935 10.1016/s0950-3536(05)80155-6

[3] Scharberg, E.A., Olsen, C., Bugert, P.: The H blood group system. Immunohematology. **32**, 112–118 (2016). 10.21307/immunohematology-2019-056 27834485

[4] Kelly, R.J., Ernst, L.K., Larsen, R.D., Bryant, J.G., Robinson, J.S., Lowe, J.B.: Molecular basis for H blood group deficiency in Bombay (O(h)) and para- Bombay individuals. Proc. Natl. Acad. Sci. U S A. **91**, 5843–5847 (1994). 10.1073/PNAS.91.13.5843 7912436 10.1073/pnas.91.13.5843 PMC44093

[5] Belo, A.I., Van Vliet, S.J., Maus, A., Laan, L.C., Nauta, T.D., Koolwijk, P., Tefsen, B., Van Die, I.: Hypoxia inducible factor 1α down regulates cell surface expression of α1,2-fucosylated glycans in human pancreatic adenocarcinoma cells. FEBS Lett. **589**, 2359–2366 (2015). 10.1016/J.FEBSLET.2015.07.035 26232512 10.1016/j.febslet.2015.07.035

[6] Che, Y., Ren, X., Xu, L., Ding, X., Zhang, X., Sun, X.: Critical involvement of the α(1,2)-fucosyltransferase in multidrug resistance of human chronic myeloid leukemia. Oncol. Rep. **35**, 3025–3033 (2016). 10.3892/OR.2016.4673 26986216 10.3892/or.2016.4673

[7] Lai, T.Y., Chen, I.J., Lin, R.J., Liao, G.S., Yeo, H.L., Ho, C.L., Wu, J.C., Chang, N.C., Lee, A.C.L., Yu, A.L.: Fucosyltransferase 1 and 2 play pivotal roles in breast cancer cells. Cell. Death Discov 5. (2019). 10.1038/S41420-019-0145-Y 10.1038/s41420-019-0145-y PMC6403244 30854233

[8] Park, S., Lim, J.M., Chun, J.N., Lee, S., Kim, T.M., Kim, D.W., Kim, S.Y., Bae, D.J., Bae, S.M., So, I., Kim, H.G., Choi, J.Y., Jeon, J.H.: Altered expression of fucosylation pathway genes is associated with poor prognosis and tumor metastasis in non–small cell lung cancer. Int. J. Oncol. **56**, 559–567 (2020). 10.3892/IJO.2019.4953 31894325 10.3892/ijo.2019.4953 PMC6959459

[9] Domino, S.E., Hurd, E.A.: LacZ expression in Fut2-LacZ reporter mice reveals estrogen-regulated endocervical glandular expression during estrous cycle, hormone replacement, and pregnancy. Glycobiology. **14**, 169–175 (2004). 10.1093/GLYCOB/CWH019 14576173 10.1093/glycob/cwh019 PMC1502365

[10] Wang, C.M., Hu, S.G., Ru, Y.F., Yao, G.X., Ma, W., Bin, Gu, Y.H., Chu, C., Wang, S.L., Zhou, Z.M., Liu, Q., Zhou, Y.C., Zhang, Y.L.: Different effects of androgen on the expression of Fut1, Fut2, Fut4 and Fut9 in male mouse reproductive tract. Int. J. Mol. Sci. **14**, 23188–23202 (2013). 10.3390/IJMS141123188 24284406 10.3390/ijms141123188 PMC3856113

[11] Newton, G.R., Lewis, S.K., Avendano, J., Williams, E.A., Ribeiro, F.R.B., Nuti, L.C., Foxworth, W.B., Ing, N.H.: Fucosyltransferase gene expression in goat endometrium during the estrous cycle and early pregnancy. Theriogenology. **132**, 118–127 (2019). 10.1016/J.THERIOGENOLOGY.2019.04.022 31022601 10.1016/j.theriogenology.2019.04.022

[12] Iwamori, M., Adachi, S., Lin, B., Tanaka, K., Aoki, D., Nomura, T.: Spermatogenesis-associated changes of fucosylated glycolipids in murine testis. Hum. Cell. **33**, 23–28 (2020). 10.1007/S13577-019-00304-X 31784953 10.1007/s13577-019-00304-x

[13] Jasper, M.J., Care, A.S., Sullivan, B., Ingman, W.V., Aplin, J.D., Robertson, S.A.: Macrophage-derived LIF and IL1B regulate alpha(1,2)fucosyltransferase 2 (Fut2) expression in mouse uterine epithelial cells during early pregnancy. Biol. Reprod. **84**, 179–188 (2011). 10.1095/BIOLREPROD.110.085399 20864644 10.1095/biolreprod.110.085399

[14] Meijerink, E., Neuenschwander, S., Fries, R., Dinter, A., Bertschinger, H., Stranzinger, G., Vögeli, P.: A DNA polymorphism influencing alpha(1,2)fucosyltransferase activity of the pig FUT1 enzyme determines susceptibility of small intestinal epithelium to Escherichia coli F18 adhesion. Immunogenetics. **52**, 129–136 (2000). 10.1007/S002510000263 11132149 10.1007/s002510000263

[15] Guillon, P., Ruvoën-clouet, N., Le Moullac-Vaidye, B., Marchandeau, S., Pendu, L.: Association between expression of the H histo-blood group antigen, alpha1,2fucosyltransferases polymorphism of wild rabbits, and sensitivity to rabbit hemorrhagic disease virus. Glycobiology. **19**, 21–28 (2009). 10.1093/GLYCOB/CWN098 18842963 10.1093/glycob/cwn098

[16] Wanninger, A., Wollesen, T.: The evolution of molluscs. Biol. Rev. **94**, 102–115 (2019). 10.1111/BRV.12439 29931833 10.1111/brv.12439 PMC6378612

[17] Morgan, J.A.T., Dejong, R.J., Snyder, S.D., Mkoji, G.M., Loker, E.S.: Schistosoma mansoni and Biomphalaria: Past history and future trends. Parasitology. **123**, 211–228 (2001). 10.1017/S0031182001007703 11769285 10.1017/s0031182001007703

[18] Robinson, T.B., Griffiths, C.L., Tonin, A., Bloomer, P., Hare, M.P.: Naturalized populations of oysters, Crassostrea gigas along the South African coast: Distribution, abundance and population structure. J. Shellfish Res. **24**, 443–450 (2005). 10.2983/0730-8000(2005)24[443:NPOOCG]2.0.CO;2

[19] Carrasco, M.F., Barón, P.J.: Analysis of the potential geographic range of the Pacific oyster Crassostrea gigas (Thunberg, 1793) based on surface seawater temperature satellite data and climate charts: The coast of South America as a study case. Biol. Invasions. **12**, 2597–2607 (2010). 10.1007/s10530-009-9668-0

[20] Iglesias, D., Rodríguez, L., Gómez, L., Azevedo, C., Montes, J.: Histological survey of Pacific oysters Crassostrea gigas (Thunberg) in Galicia (NW Spain). (2012). 10.1016/j.jip.2012.08.015 10.1016/j.jip.2012.08.015 22985902

[21] Clegg, T.A., Morrissey, T., Geoghegan, F., Martin, S.W., Lyons, K., Ashe, S., More, S.J.: Risk factors associated with increased mortality of Farmed Pacific oysters in Ireland during 2011. Prev. Vet. Med. **113**, 257–267 (2014). 10.1016/J.PREVETMED.2013.10.023 24290496 10.1016/j.prevetmed.2013.10.023

[22] Dridi, S., Romdhane, M.S., Elcafsi, M.: Gametogenic cycle of Crassostrea gigas in contrasting Mediterranean habitats: Marine (Gulf of Tunis) and continental (bizert lagoon) culture sites. J. Biol. Res. (Thessalonike Greece). **21** (2014). 10.1186/2241-5793-21-13 10.1186/2241-5793-21-13 PMC4390001 25984496

[23] Jonathan, M.P., Muñoz-Sevilla, N.P., Góngora-Gómez, A.M., Varela, L., Sujitha, R.G., Escobedo-Urías, S.B., Rodríguez-Espinosa, D.C., Villegas, P.F.C.: Bioaccumulation of trace metals in farmed pacific oysters Crassostrea gigas from SW Gulf of California coast. Mexico Chemosphere. **187**, 311–319 (2017). 10.1016/J.CHEMOSPHERE.2017.08.098 28858712 10.1016/j.chemosphere.2017.08.098

[24] Kijas, J.W., Gutierrez, A.P., Houston, R.D., McWilliam, S., Bean, T.P., Soyano, K., Symonds, J.E., King, N., Lind, C., Kube, P.: Assessment of genetic diversity and population structure in cultured Australian Pacific oysters. Anim. Genet. **50**, 686–694 (2019). 10.1111/AGE.12845 31518019 10.1111/age.12845

[25] Corporeau, C., Huvet, A., Pichereau, V., Delisle, L., Quéré, C., Dubreuil, C., Artigaud, S., Brenner, C., Cunha-De Padua, M.M., Mazure, N.: The oyster Crassostrea gigas, a new model against cancer. Med. Sci. (Paris). **35**, 463–466 (2019). 10.1051/MEDSCI/2019079 31115329 10.1051/medsci/2019079

[26] McLeod, C., Polo, D., Le Saux, J.C., Le Guyader, F.S.: Depuration and relaying: A review on potential removal of Norovirus from oysters. Compr. Rev. Food Sci. Food Saf. **16**, 692–706 (2017). 10.1111/1541-4337.12271 33371561 10.1111/1541-4337.12271

[27] Zheng, Q., Van Die, I., Cummings, R.D.: Molecular cloning and characterization of a novel alpha 1,2-fucosyltransferase (CE2FT-1) from Caenorhabditis elegans. J. Biol. Chem. **277**, 39823–39832 (2002). 10.1074/JBC.M207487200 12163507 10.1074/jbc.M207487200

[28] Zheng, Q., Van die, I., Cummings, R.D.: A novel alpha1,2-fucosyltransferase (CE2FT-2) in Caenorhabditis elegans generates H-type 3 glycan structures. Glycobiology. **18**, 290–302 (2008). 10.1093/GLYCOB/CWN007 18252778 10.1093/glycob/cwn007

[29] Mulder, H., Schachter, H., Thomas, J.R., Halkes, K.M., Kamerling, J.P., Vliegenthart, J.F.G.: Identification of a GDP-Fuc:Galβ1-3GalNAc-R (fuc to gal) α1–2 fucosyltransferase and a GDP-Fuc:Galβ1-4GlcNAc (fuc to GlcNAc) α1–3 fucosyltransferase in connective tissue of the snail Lymnaea stagnalis. Glycoconj. J. **13**, 107–113 (1996). 10.1007/BF01049686/METRICS. 8785481 10.1007/BF01049686

[30] Gui, B., Yao, L., Qu, M., Zhang, W., Li, M., Jiang, Y., Wang, L.: Cloning, expression, and functional characterization of FUT1, a Key Gene for Histo-Blood Group Antigens Synthesis in Crassostrea gigas. Curr. Issues Mol. Biol. **45**, 4200–4213 (2023). 10.3390/CIMB45050267 37232736 10.3390/cimb45050267 PMC10217650

[31] Zemkollari, M., Blaukopf, M., Grabherr, R., Staudacher, E.: Expression and characterisation of the First snail-derived UDP-Gal: Glycoprotein-N-acetylgalactosamine β-1,3-Galactosyltransferase (T-Synthase) from Biomphalaria glabrata. Molecules. **28**, 552 (2023). 10.3390/molecules28020552 36677618 10.3390/molecules28020552 PMC9865085

[32] Stepan, H., Staudacher, E.: Optimization of monosaccharide determination using anthranilic acid and 1-phenyl-3-methyl-5-pyrazolone for gastropod analysis. Anal. Biochem. **418**, 24–29 (2011). 10.1016/J.AB.2011.07.005 21802397 10.1016/j.ab.2011.07.005 PMC3169793

[33] Hase, S., Ibuki, T., Ikenaka, T.: Reexamination of the pyridylamination used for fluorescence labeling of oligosaccharides and its application to glycoproteins. J. Biochem. **95**, 197–203 (1984). 10.1093/OXFORDJOURNALS.JBCHEM.A134585 6706908 10.1093/oxfordjournals.jbchem.a134585

[34] Ruprecht, C., Bartetzko, M.P., Senf, D., Lakhina, A., Smith, P.J., Soto, M.J., Oh, H., Yang, J.Y., Chapla, D., Silva, V., Clausen, D., Hahn, M.H., Moremen, M.G., Urbanowicz, K.W., Pfrengle, B.R.: A glycan array-based assay for the identification and characterization of Plant glycosyltransferases. Angew. Chemie - Int. Ed. **59**, 12493–12498 (2020). 10.1002/anie.202003105 10.1002/anie.202003105 PMC7383710 32396713

[35] Andersen, M.C.F., Boos, I., Marcus, S.E., Kračun, S.K., Rydahl, M.G., Willats, W.G.T., Knox, J.P., Clausen, M.H.: Characterization of the LM5 pectic galactan epitope with synthetic analogues of β-1,4-d-galactotetraose. Carbohydr. Res. **436**, 36–40 (2016). 10.1016/J.CARRES.2016.10.012 27855335 10.1016/j.carres.2016.10.012

[36] Andersen, M.C.F., Boos, I., Ruprecht, C., Willats, W.G.T., Pfrengle, F., Clausen, M.H.: Synthesis and application of branched type II arabinogalactans. J. Org. Chem. **82**, 12066–12084 (2017). 10.1021/ACS.JOC.7B01796 29120180 10.1021/acs.joc.7b01796

[37] Andersen, M.C.F., Kračun, S.K., Rydahl, M.G., Willats, W.G.T., Clausen, M.H.: Synthesis of b-1,4-Linked Galactan Side-Chains of Rhamnogalacturonan I. Chem. Eur. J. **22** (2016). 10.1002/chem.201602197 10.1002/chem.201602197 27305141

[38] Bartetzko, M.P., Schuhmacher, F., Hahm, H.S., Seeberger, P.H., Pfrengle, F.: Automated Glycan Assembly of oligosaccharides related to Arabinogalactan proteins. Org. Lett. **17** (2015). 10.1021/acs.orglett.5b02185 10.1021/acs.orglett.5b02185 26295743

[39] Bartetzko, M.P., Schuhmacher, F., Seeberger, P.H., Pfrengle, F.: Determining substrate specificities of β1,4-Endogalactanases using Plant Arabinogalactan oligosaccharides synthesized by Automated Glycan Assembly. J. Org. Chem. **82** (2017). 10.1021/acs.joc.6b02745 10.1021/acs.joc.6b02745 28075586

[40] Dallabernardina, P., Ruprecht, C., Smith, P.J., Hahn, M.G., Urbanowicz, B.R., Pfrengle, F.: Automated glycan assembly of galactosylated xyloglucan oligosaccharides and their recognition by plant cell wall glycan-directed antibodies. Org. Biomol. Chem. **15**, 9996–10000 (2017). 10.1039/C7OB02605F 29177276 10.1039/c7ob02605f PMC6082630

[41] Breton, C., Oriol, R., Imberty, A.: Conserved structural features in eukaryotic and prokaryotic fucosyltransferases. Glycobiology. **8**, 87–94 (1998). 10.1093/glycob/8.1.87 9451017 10.1093/glycob/8.1.87

[42] Zeng, X., Morimoto, S., Murata, T., Usui, T.: Regioselectivity of a-L-fucopyranosyloligosaccharide formation by the α-L-Fucosidase from Porcine Liver. J. Appl. Glycosci. **46**, 241–247 (1999). 10.5458/jag.46.241

[43] Larsen, R.D., Ernst, L.K., Nair, R.P., Lowe, J.B.: Molecular cloning, sequence, and expression of a human GDP-L-fucose:β-D-galactoside 2-α-L-fucosyltransferase cDNA that can form the H blood group antigen. Proc. Natl. Acad. Sci. U S A. **87**, 6674–6678 (1990). 10.1073/pnas.87.17.6674 2118655 10.1073/pnas.87.17.6674 PMC54599

[44] Cohney, S., Mouhtouris, E., McKenzie, I.F.C., Sandrin, M.S.: Molecular cloning and characterization of the pig secretor type α-1,2-fucosyltransferase (FUT2). Int. J. Mol. Med. **3**, 199–207 (1999). 10.3892/ijmm.3.2.199 9917530 10.3892/ijmm.3.2.199

[45] Morozov, V., Hanisch, F.G., Wegner, K.M., Schroten, H.: Pandemic GII.4 Sydney and epidemic GII.17 Kawasaki308 noroviruses display distinct specificities for histo-blood group antigens leading to different transmission vector dynamics in Pacific oysters. Front. Microbiol. **9**, 417400 (2018). 10.3389/FMICB.2018.02826/BIBTEX 10.3389/fmicb.2018.02826 PMC6278567 30542329

[46] Le Guyader, F.S., Loisy, F., Atmar, R.L., Hutson, A.M., Estes, M.K., Ruvoën-Clouet, N., Pommepuy, M., Le Pendu, J.: Norwalk virus-specific binding to oyster digestive tissues. Emerg. Infect. Dis. **12**, 931–936 (2006). 10.3201/EID1206.051519 16707048 10.3201/eid1206.051519 PMC2596755

[47] Tian, P., Bates, A.H., Jensen, H.M., Mandrell, R.E.: Norovirus binds to blood group A-like antigens in oyster gastrointestinal cells. Lett. Appl. Microbiol. **43**, 645–651 (2006). 10.1111/J.1472-765X.2006.02010.X 17083711 10.1111/j.1472-765X.2006.02010.x

[48] Tian, P., Engelbrektson, A.L., Jiang, X., Zhong, W., Mandrell, R.E.: Norovirus recognizes histo-blood group antigens on gastrointestinal cells of clams, mussels, and oysters: A possible mechanism of bioaccumulation. J. Food Prot. **70**, 2140–2147 (2007). 10.4315/0362-028X-70.9.2140 17900094 10.4315/0362-028x-70.9.2140

