# Supplementary material for: Cloning, expression and characterisation of a novel mollusc α-1,2-Fucosyltransferase from Crassostrea gigas (CgFUT2)
Source: Glycoconj J. 2024 Aug 20;41(4-5):255–65. doi: 10.1007/s10719-024-10162-x (PMC11522050; doi:10.1007/s10719-024-10162-x)
Supplement: Supplementary file 1 — Supplementary Material 1 [file 10719_2024_10162_MOESM1_ESM.pdf]

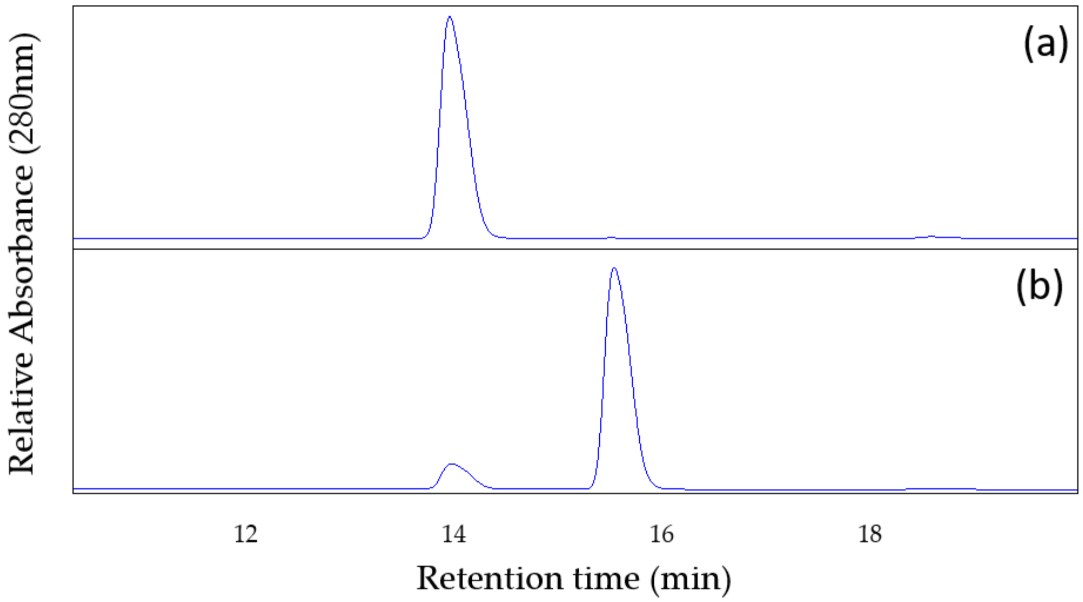

Fig. S1 HPLC analysis of the transfer of Fuc to pNP-β-lactose. (a) Standard pNP-β-lactose and (b) pNP-β-lactose incubated with *Cg*FUT2

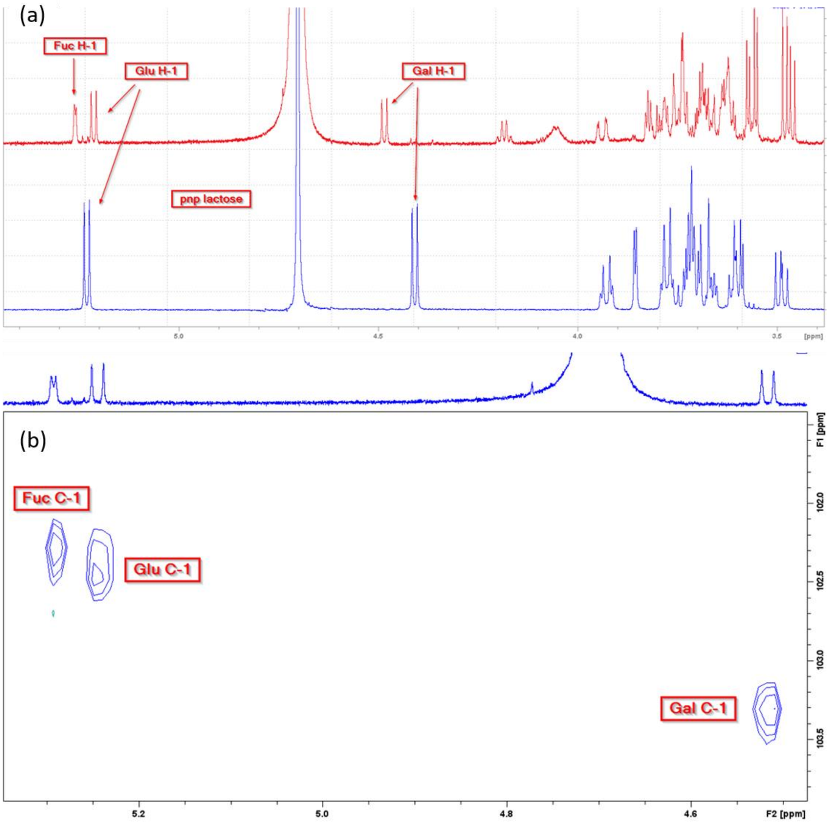

Fig. S2 (a) ^{1}H NMR comparison of pNP-lactose (blue, lower graph) and obtained transfer product (red, upper graph). (b) HSQC of the anomeric region with the newly introduced Fuc C-1 and the already present Gal and Glc C-1

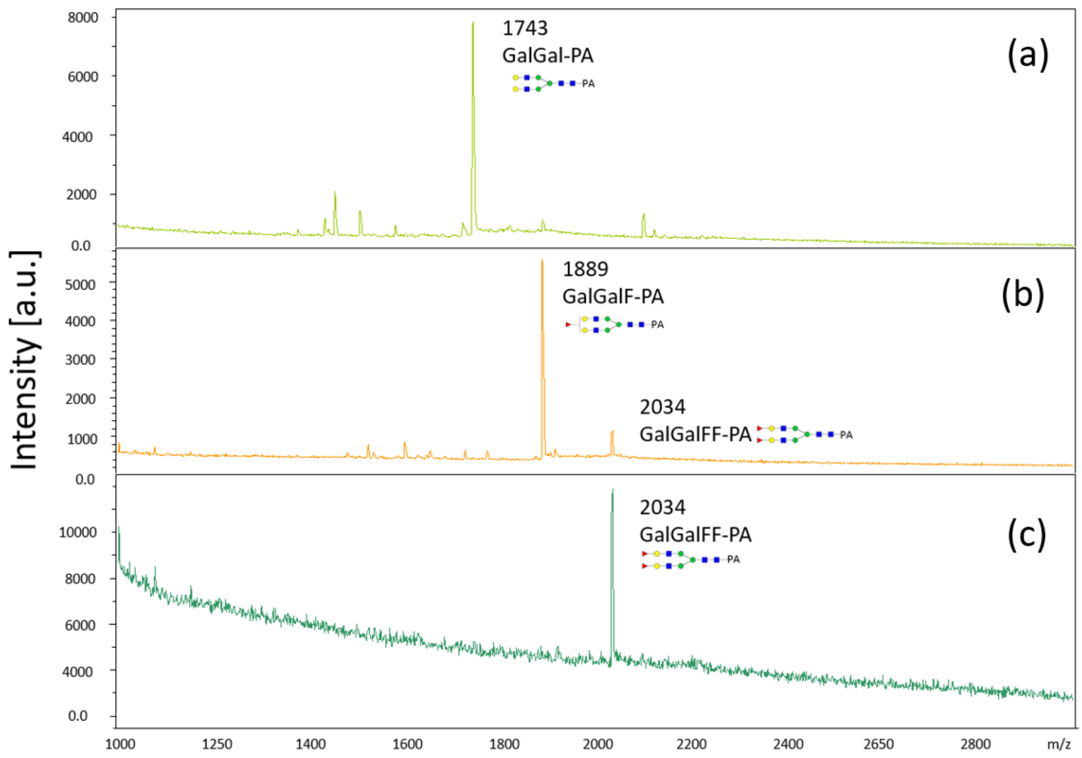

Fig. S3 MALDI-TOF analysis of the transfer of Fuc to terminal galactoses of GalGal-PA. (a) GalGal-PA substrate, (b) one fucose residue added and (c) two fucose residues added

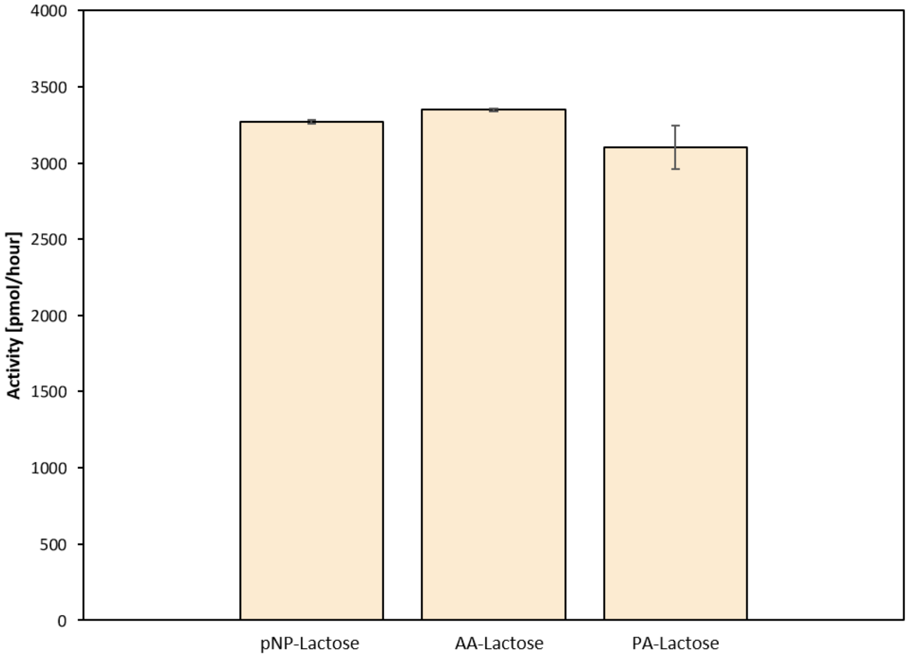

Figure S4: Effects of different sugar labels on the activity of *Cg*FUT2

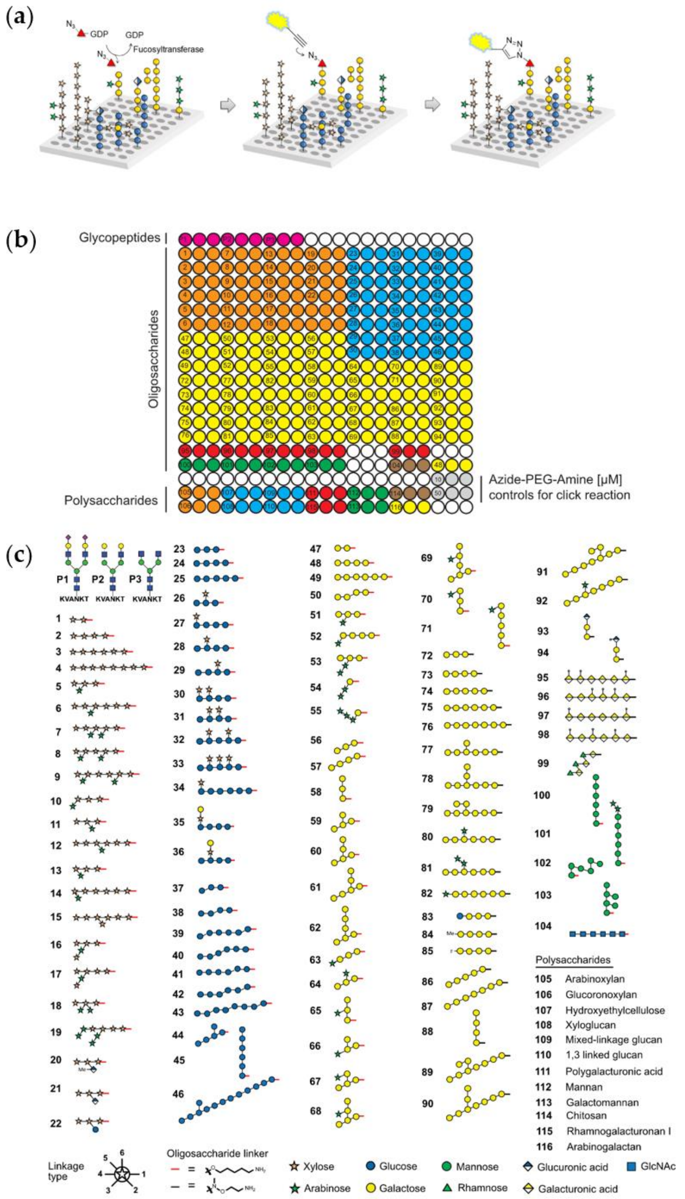

Figure S5: Glycosyltransferase glycan microarray principle, printing pattern, and printed compounds. (a) Principle of the glycan microarray for the analysis of glycosyltransferases. (b) Printing pattern of the array. Each compound is printed in triplicates at concentration of 200 µM for oligosaccharides and 200 µg/ml for polysaccharides. (c) Complete list of printed compounds, including the structures of the oligosaccharides. Oligosaccharides were chemically synthesized, the glycopeptides were purified from egg yolk. Figure is modified from [34]

No.	AV	SD	No.	AV	SD	No.	AV	SD	No.	AV	SD	No.	AV	SD
Glycopeptides			Glucans			Galactans			Galactans			Other		
P1	218	3	23	4	2	47	64	5	71	3	1	95	9	1
P2	2477	94	24	5	1	48	468	30	72	157	13	96	6	2
P3	21	2	25	4	0	49	484	40	73	314	22	97	6	1
Xylans			26	2	1	50	77	8	74	320	12	98	9	1
1	1	1	27	1	1	51	4	1	75	357	7	99	10	2
2	1	0	28	3	2	52	20	4	76	351	6	100	1	1
3	2	0	29	4	1	53	13	2	77	16	3	101	2	2
4	2	1	30	0	1	54	2	1	78	25	1	102	0	1
5	1	0	31	2	2	55	3	1	79	22	2	103	1	0
6	0	0	32	3	0	56	546	21	80	24	2	104	1	1
7	2	1	33	2	1	57	532	21	81	22	3	Polysaccharides		
8	3	0	34	7	1	58	47	1	82	40	3	105	2	0
9	2	1	35	7	2	59	590	28	83	6	1	106	2	1
10	1	1	36	400	24	60	317	3	84	754	22	107	22	6
11	1	1	37	38	4	61	466	14	85	11	1	108	7	1
12	0	0	38	12	1	62	200	18	86	383	24	109	1	1
13	0	1	39	2	0	63	37	4	87	445	6	110	0	1
14	1	0	40	11	1	64	851	43	88	20	2	111	2	1
15	1	0	41	8	1	65	35	2	89	396	18	112	1	1
16	1	1	42	22	1	66	46	4	90	299	7	113	13	13
17	2	1	43	7	3	67	654	41	91	284	10	114	19	30
18	0	1	44	0	1	68	380	23	92	395	24	115	97	10
19	1	1	45	3	1	69	272	14	93	24	30	116	8	7
20	2	0	46	4	1	70	6	1	94	7	2	Azides		
21	2	1				71	3	1				Az10	2135	134
22	3	0										Az50	2038	227

Table S1: Fluorescence values for the acceptor substrates on the array. No. ; Number of the compound (see Figure S4). AV: Average fluorescence of the triplicates. SD: standard deviation
